# RNA modifications in intestinal macrophages: Implications for gut immunity and inflammation

**DOI:** 10.1016/j.gendis.2025.101881

**Published:** 2025-10-17

**Authors:** Manqiqige Su, Jiyuan Fan, Hua-Bing Li

**Affiliations:** a Department of Oncology, Laboratory of Immunity, Inflammation & Cancer, The First Affiliated Hospital of Chongqing Medical University, Chongqing 400016, China; b Institute for Immunology and Pathogenesis (IIP), Chongqing Medical University, Chongqing 400016, China; c Medical Center on Aging, Center for Immune-Related Diseases at Shanghai Institute of Immunology, Ruijin Hospital, Shanghai Jiao Tong University School of Medicine, Shanghai 200025, China; d Department of Immunobiology, Yale University School of Medicine, New Haven, CT 06520, USA

**Keywords:** Epitranscriptomics, Inflammatory bowel disease, Intestinal macrophage, Macrophage plasticity, RNA modification

## Abstract

Intestinal macrophages are critical regulators of mucosal immunity, playing essential roles in microbial surveillance, barrier maintenance, and tissue repair. As highly responsive immune cells, they integrate diverse environmental cues to dynamically adapt to their functional states. In recent years, RNA modifications have emerged as a key layer of post-transcriptional regulation, orchestrating macrophage development, polarization, and immunometabolic programming. This review focuses on the role of epitranscriptomic regulation in shaping the plasticity of intestinal macrophages, systematically summarizing how RNA modifications influence their responses to inflammatory stimuli, microbial signals, and intercellular communication. We further highlight the regulatory potential of RNA modifications in gut immune homeostasis and inflammatory diseases, providing a comprehensive framework for understanding RNA-mediated immune regulation and a forward-looking perspective on targeting these pathways in intestinal disorders.

## Introduction

Intestinal macrophages are central players in maintaining mucosal immune homeostasis, defending against pathogens, and coordinating tissue repair. These cells arise not only from long-lived, self-renewing embryonically derived resident macrophages,^1^ but also from continuously recruited Lymphocyte antigen 6 complex locus C-positive (Ly6C^+^) monocytes that differentiate into intestinal mononuclear phagocytes throughout adulthood.^2^ This dual developmental origin distinguishes intestinal macrophages from those in tissues such as the heart and lung, where resident macrophages are typically maintained independently of bone marrow input in adulthood.^3^^,^^4^

The intestinal microenvironment comprises multiple cellular compartments—epithelial, immune, and stromal (mesenchymal) cells—alongside signaling components, such as microbial metabolites, neuropeptides, and cytokines, which collectively maintain gastrointestinal immune homeostasis.^5^, ^6^, ^7^, ^8^, ^9^ Compared with other tissues, the intestinal environment presents unique challenges, requiring immune cells to simultaneously tolerate commensal microbiota and dietary antigens while responding to inflammatory stimuli.^10^^,^^11^ As a result, intestinal macrophages exhibit remarkable phenotypic and functional plasticity. This complexity demands highly adaptable regulatory mechanisms, positioning intestinal macrophages as responsive sensors and integrators of environmental cues.

In recent years, RNA modifications—chemical modifications to RNA that modulate gene expression post-transcriptionally—have emerged as critical regulators of immune cell fate, function, and polarization. Studies by our group and others have shown that RNA modifications, including N^6^-methyladenosine (m^6^A), N1-methyladenosine (m^1^A), and 5-methylcytosine (m^5^C), regulate inflammatory responses, antigen presentation, and metabolic reprogramming in T cells, dendritic cells, and tumor-associated macrophages.^12^, ^13^, ^14^, ^15^ Within the intestinal environment, our recent work also demonstrated that intestinal epithelial cell renewal, differentiation, and apoptosis are governed by epigenetic mechanisms.^16^^,^^17^ Furthermore, studies in inflammatory bowel disease and colorectal cancer have shown that dysbiosis of gut microbiota can modulate immune gene expression through epigenetic regulation, ultimately leading to immune dysregulation.^18^ Emerging (unpublished) data from our group further suggest that m^6^Am (N^6^,2′-O-dimethyladenosine) RNA methylation, catalyzed by phosphorylated CTD-interacting factor 1 (*Pcif1*), may mediate the crosstalk between intestinal macrophages and enteric neurons through extracellular matrix remodeling, revealing an additional layer of post-transcriptional regulation in gut immunity.

However, whether and how these RNA modifications regulate the differentiation and activation of intestinal macrophages, particularly during the transition from blood monocytes to tissue-resident cells and under pathological conditions, remains largely unexplored. In this review, we aim to systematically summarize the current understanding of RNA modification-mediated regulation of macrophage function, with a particular focus on intestinal mononuclear phagocytes. We discuss how RNA modifications may influence intestinal immune homeostasis and contribute to disease pathogenesis, and highlight their potential as promising therapeutic targets for inflammatory and neoplastic disorders of the gut.

## Intestinal macrophage-monocytes

**Figure 1:**
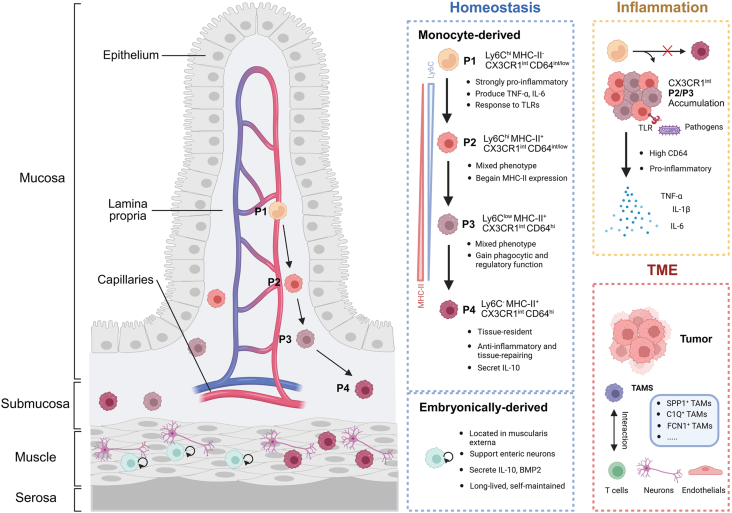
Differentiation and functional plasticity of intestinal macrophages across tissue states. Monocyte-derived macrophages differentiate stepwise from Ly6C^hi^ monocytes (P1) to MHC-II^+^ tissue-resident macrophages (P4) in the lamina propria under homeostasis, accompanied by changes in surface markers and functional states. Inflammation disrupts this progression, leading to P2/P3 accumulation and enhanced pro-inflammatory cytokine production. Embryonically-derived macrophages reside in the muscularis externa, support enteric neurons, and maintain anti-inflammatory functions. In the tumor microenvironment, monocyte-derived cells give rise to heterogeneous TAM subsets (*e.g.*, SPP1^+^, C1Q^+^, FCN1^+^) that interact with stromal and immune cells to shape tumor immunity. TAMs, tumor-associated macrophages; TME, tumor microenvironment; TLR, toll-like receptor.

Intestinal macrophages originate from both long-lived, self-renewing embryonic precursors and continuously replenished Ly6C^+^ circulating monocytes that differentiate into monocyte-derived macrophages in the lamina propria. In mice, these recruited cells in mice undergo a well-characterized maturation process known as the “monocyte waterfall”, transitioning from recently extravasated population 1 (P1) cells to fully differentiated population 4 (P4) macrophages over 5–7 days (Fig. 1). This progression involves down-regulation of Ly6C and C–C motif chemokine receptor 2 (CCR2) and up-regulation of major histocompatibility complex class II (MHC-II), C-X3-C motif chemokine receptor 1 (CX3CR1), cluster of differentiation 11b (CD11b), CD11c, and CD64^19^, ^20^, ^21^ Specifically, P1 cells are Ly6C^hi^ MHC-II^-^ CX3CR1^int^, while mature P4 macrophages are Ly6C^lo^ MHC-II^+^ CX3CR1^hi^ CD11b^+^ CD11c^+^, exhibiting high phagocytic activity and anti-inflammatory potential.^22^^,^^23^ Additional subset distinctions can be made based on T-cell/transmembrane immunoglobulin and mucin domain containing 4 (TIM4) and CD4 expression, identifying short-lived TIM4^-^ CD4^-^ and long-lived TIM4^+^ CD4^+^ macrophages.^24^ In humans, a similar differentiation trajectory has been described, with CD14^++^ CD16^-^ classical monocytes progressively acquiring CD64, CX3CR1, and CD11b during tissue residency.^25^^,^^26^

These mature macrophages are key to maintaining intestinal homeostasis, characterized by tolerance to commensal microbes and the production of immunoregulatory cytokines such as interleukin (IL)-10, prostaglandin E2 (PGE_2_), and transforming growth factor-beta (TGF-β).^27^ However, under inflammatory conditions such as inflammatory bowel disease or bacterial infection, monocyte differentiation may be arrested, leading to an accumulation of CX3CR1^int^ inflammatory intermediates that fail to fully acquire tolerogenic functions and instead produce elevated levels of tumor necrosis factor-alpha (TNF-α), IL-1β, and IL-6. In infectious models, monocytes rapidly infiltrate and differentiate into proinflammatory macrophages that amplify immune responses.^28^ Moreover, emerging evidence suggests that stromal signals, including colony-stimulating factor 1 (CSF1)/colony-stimulating factor 1 receptor (CSF1R) and granulocyte macrophage-colony-stimulating factor (GM-CSF) from platelet-derived growth factor receptor alpha-positive (PDGFRA^+^) fibroblasts, contribute to monocyte differentiation trajectories in both homeostatic and disease contexts.^29^^,^^30^ In addition, intestinal microbiota can influence monocyte-derived macrophage activation and cytokine output. Microbial cues have been shown to enhance IL-1β and IL-6 production in Ly6C^+^ macrophages, contributing to the inflammatory milieu that drives colitis-associated tumorigenesis.^31^

In colorectal cancer, monocyte-derived macrophages are shaped by a complex network of cues from the tumor microenvironment, including tumor cell-derived signals, cytokines and chemokines, metabolic factors, extracellular matrix components, and interactions with other immune cells. These factors collectively drive spatial and temporal heterogeneity among macrophage populations. Single-cell RNA sequencing analyses have revealed that intestinal macrophages in the tumor microenvironment differentiate into diverse tumor-associated macrophage subsets with distinct phenotypic and functional characteristics.^32^^,^^33^

Collectively, these findings underscore the highly plastic and context-dependent nature of intestinal macrophages, whose differentiation and function are tightly regulated by tissue microenvironmental cues. Recent evidence further suggests that such environmental signals may influence macrophage fate not only via transcriptional networks but also through post-transcriptional mechanisms—including RNA modifications—that fine-tune gene expression in response to dynamic local stimuli.

## Functional implications of RNA modifications in monocyte-to-macrophage plasticity

**Figure 2:**
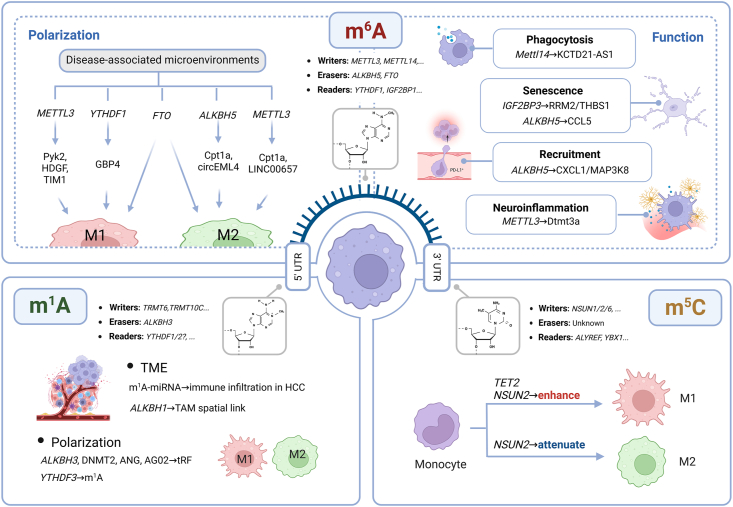
RNA modifications in macrophage polarization and function. m^6^A modification, installed by *METTL3/METTL14* and interpreted by *YTHDF* and IGF2BP proteins, influences macrophage polarization in response to disease-associated microenvironments and regulates processes, including phagocytosis, senescence, immune recruitment, and neuroinflammation. m^1^A modification, catalyzed by *TRMT6/TRMT10C* and removed by *ALKBH3*, has been linked to tumor-associated macrophage localization and immune infiltration via miRNA-associated pathways. m^1^A-associated factors, such as DNMT2, ANG, and AGO2, contribute to tRNA-derived fragment-mediated regulation of macrophage polarization. m^5^C modification, regulated by the *NSUN* family and *TET2*, promotes M2 polarization and attenuates M1 responses through transcriptional reprogramming. m^6^A, N^6^-methyladenosine; m^1^A, N^1^-methyladenosine; m^5^C, 5-methylcytosine; TAM, tumor-associated macrophage; tRF, tRNA-derived fragment; UTR, untranslated region; TME, tumor microenvironment.

As a pivotal post-transcriptional regulatory mechanism, RNA modifications have garnered increasing attention for their roles in modulating immune cell function. Macrophages, which serve as critical mediators bridging innate and adaptive immunity, rely heavily on RNA modifications to fine-tune their polarization, cytokine production, phagocytic capacity, and metabolic reprogramming (Fig. 2). Among the various RNA modifications identified in mammals, the most extensively studied include m^6^A, m^1^A, and m^5^C. These modifications are dynamically regulated by three classes of proteins: writers (*e.g.*, *METTL3*, *NSUN2*), which catalyze the installation of methyl groups; erasers (*e.g.*, *FTO*, *ALKBH5*, *ALKBH3*), which remove these modifications; and readers (*e.g.*, the *YTHDF* protein family), which recognize and interpret the modified bases to execute downstream functions. In the following sections, we focus on the functional relevance of these three major types of RNA modifications in regulating monocyte and macrophage biology.

## Regulatory dynamics of m^6^A modification in macrophage immunity

m^6^A is a chemical modification in which a methyl group is added to the nitrogen-6 position of adenosine within RNA molecules. It represents the most prevalent, abundant, and evolutionarily conserved internal post-transcriptional modification in eukaryotic RNA. In messenger RNA (mRNA), m^6^A modification is reversible and dynamically regulated by a set of enzymes: *writers* such as methyltransferase-like 3 (*METTL3*) and *METTL14* catalyze methylation; *erasers* like alkane monooxygenase homolog 5 (ALKBH5) and fat mass and obesity-associated (FTO) remove the methyl group; and *readers*, including the YTH domain family proteins (*YTHDF1*, *YTHDF2*, *YTHDF3*, and *YTHDC1*), recognize m^6^A-modified sites and mediate downstream regulatory outcomes.^34^ These proteins collectively determine the fate of mRNA by modulating its stability, splicing, nuclear export, localization, and translational efficiency. m^6^A modifications are enriched in coding sequences (CDS), 3′ untranslated regions (3′ UTRs), long introns, and regions near stop codons. This modification is widely distributed in both coding and non-coding RNAs and plays critical roles in regulating immune cell development and function, contributing to cellular differentiation, organismal development, and the pathogenesis of various diseases.^35^

### Bidirectional plasticity of m^6^A modification in monocyte-macrophage polarization

Macrophage m^6^A modifications are multifaceted, exerting both pro-inflammatory and anti-inflammatory effects. Rather than unidirectionally promoting M1 (type I) or M2 (type II) polarization, m^6^A acts in a context-dependent manner, with its functional outcomes shaped by the pathological state, the tissue microenvironment, and the composition of m^6^A regulatory machinery.^36^ Depending on the local microenvironment, inflammatory cues, cellular metabolic status, and the expression patterns of methylation-related enzymes, m^6^A modifications can either promote pro-inflammatory M1 polarization or facilitate anti-inflammatory M2 phenotypes.^37^

Expression of core methyltransferases *METTL3* and *METTL14* is often up-regulated in M1-polarized macrophages, contributing to elevated global m^6^A methylation levels and reinforcing pro-inflammatory transcriptional programs.^38^^,^^39^ In atherosclerosis models, the *Mettl3/Ythdf2* axis enhances the stability of protein tyrosine kinase 2 (Pyk2) mRNA, promoting reactive oxygen species production and inflammatory cytokine secretion, thereby driving M1 polarization and exacerbating local inflammation.^40^ Similarly, *Mettl3*-mediated m^6^A methylation of hepatoma-derived growth factor (HDGF) augments glycolytic flux, reinforcing the pro-inflammatory macrophage phenotype.^41^ Another study demonstrated that *METTL3* modulates TIM1 expression via m^6^A modification, which in turn is recognized by insulin-like growth factor 2 mRNA binding protein 2 (*IGF2BP2*) to induce M1 polarization and promote IL-1β and TNF-α expression.^42^

Although most studies report *METTL3* as a driver of M1 polarization, several studies have also shown that *METTL3* can promote M2-like macrophage polarization. Trimethylamine N-oxide (TMAO) can promote type II macrophage polarization by enhancing m^6^A modification of carnitine palmitoyltransferase 1A (Cpt1a) via *METTL3*, aggravating inflammation in the aortic valve.^43^ In allergic asthma, conditional deletion of *Mettl3* in myeloid cells disrupts macrophage homeostasis by enhancing Th2 responses and promoting M2 polarization.^44^ *METTL3* was also found to promote breast cancer progression via m^6^A-dependent exosomal LINC00657, which induces M2 macrophage polarization in the tumor microenvironment.^45^

Conversely, *ALKBH5* has been shown to enhance M2 polarization in several disease models. In tumor-associated macrophages within colorectal cancer, *ALKBH5* removes m^6^A marks from CPT1A mRNA, increasing its stability and expression, thereby promoting fatty acid oxidation and M2 polarization.^46^ In lung cancer models, *ALKBH5* regulates the circRNA echinoderm microtubule-associated protein-like 4 (circEML4)/miRNA/suppressor of cytokine signaling 2 (SOCS2) axis, supporting the M2-like function of tumor-associated macrophages and facilitating immune evasion in non-small cell lung cancer.^47^ *FTO*, another demethylase, has been implicated in modulating both M1 and M2 activation states, highlighting the dynamic balance orchestrated by m^6^A demethylation in polarization plasticity.^48^ Furthermore, forkhead box M1 (FOXM1)-activated *IGF2BP3* enhances ribonucleotide reductase M2 (RRM2) mRNA stability in an m^6^A-dependent manner, inhibiting ferroptosis and promoting M2 polarization in hepatocellular carcinoma.^49^

Exogenous stimuli also influence macrophage polarization via m^6^A pathways. Bacteroides fragilis toxin inhibits *METTL3* expression and its associated m^6^A activity, enhancing pro-inflammatory responses and exacerbating inflammatory bowel disease progression.^50^ *YTHDF1* can facilitate the translation of guanylate binding protein 4 (GBP4), promoting M1 polarization in acute lung injury,^51^ while m^6^A-regulated circRNA-miRNA axes, such as circ_0066715/miR-486-5p/ETS proto-oncogene 1 (ETS1) axis, are closely associated with M2 polarization in rheumatoid arthritis.^52^ Notably, in cardiac fibrosis, *ALKBH5* modulates m^6^A methylation of IL-11 mRNA to drive the transdifferentiation of cardiac macrophages into myofibroblast-like cells, indicating a pivotal role in pathological remodeling.^53^ Finally, in myocardial infarction and fibrotic conditions, m^6^A facilitates monocyte-to-repair macrophage or myofibroblast transition. Noncoding RNAs such as metastasis-associated lung adenocarcinoma transcript 1 (Malat1) can modulate peroxisome proliferator-activated receptor gamma (PPARγ) expression and macrophage-associated inflammation through epigenetic mechanisms in myocardial infarction, suggesting a potential interplay with m^6^A-mediated immune remodeling.^54^

In summary, m^6^A modifications serve as context-dependent regulators of monocyte–macrophage polarization. Through dynamic interplay among writers, erasers, and readers, they orchestrate the balance between pro-inflammatory and anti-inflammatory phenotypes. This tunable epitranscriptomic system offers promising therapeutic avenues for inflammatory diseases and tumor immunity.

### Multifaceted regulation of macrophage function by m^6^A modification

Beyond polarization, m^6^A modifications govern multiple layers of macrophage function, including phagocytosis, autophagy, glycolysis, senescence, tissue repair, and intercellular communication, thereby playing vital roles in maintaining tissue homeostasis and modulating disease progression.^55^ m^6^A-modulated mRNA stability, particularly via *IGF2BP1*, regulates the expression of RRM2 and thrombospondin 1 (THBS1), contributing to resistance against ferroptosis and the glycolytic reprogramming of tumor-associated macrophages, ultimately promoting tumor progression and fibrosis.^49^^,^^56^ In hepatocellular carcinoma, RNA-binding motif protein 15 (RBM15) and *ALKBH5* regulate the recruitment of programmed death-ligand 1-positive (PD-L1^+^) macrophages via C-X-C motif chemokine ligand 11 (CXCL11) and mitogen-activated protein kinase 8 (MAP3K8) pathways, facilitating the formation of immunosuppressive microenvironments.^57^

Moreover, m^6^A participates in macrophage senescence and immune clearance. *ALKBH5* modulates C–C motif chemokine ligand 5 (CCL5) methylation to orchestrate senescence-associated transcriptional programs in atherosclerotic macrophages.^58^ Meanwhile, potassium channel tetramerization domain containing 21 (KCTD21)-antisense RNA 1 (AS1) m^6^A modification regulates CD47 expression and TOR signaling pathway regulator (TIPR)-mediated autophagy, influencing macrophage phagocytic capacity.^59^ In Alzheimer's disease models, *METTL3* knockout improves the neuroinflammatory pathology by enhancing microglial function.^60^ Similarly, in metabolic dysfunction-associated steatotic liver disease and obesity, m^6^A-directed activation of myeloid cells shapes metabolic inflammation and immune homeostasis.^61^

Single-cell transcriptomics has further revealed that infection-specific m^6^A landscapes in macrophages dynamically reshape functional responses. In *Treponema pallidum* infection, m^6^A profiling unveiled diverse transcriptomic programs aligned with infection-related pathways.^62^ In diseases like emphysema and colorectal cancer, m^6^A-modified exosomal miRNAs mediate macrophage-epithelial crosstalk, regulating inflammatory signaling and immunotherapy responses.^63^^,^^64^

Altogether, m^6^A establishes a complex regulatory network spanning metabolic rewiring, structural remodeling, intercellular signaling, and immune modulation. It confers macrophages with remarkable functional heterogeneity and adaptability, expanding their pathophysiological roles and presenting new opportunities for targeted interventions in disease-specific contexts.

## m^5^C modification and its immunomodulatory potential in macrophages

m^5^C is a prevalent RNA modification primarily catalyzed by the Nol1/Nop2/SUN domain (NSUN) family of methyltransferases and is widely distributed in both mRNA and non-coding RNAs. m^5^C plays critical roles in regulating RNA stability, nuclear export, and cellular stress responses. However, its specific functions in immune cells remain largely unexplored.

Emerging studies have revealed that *NSUN2* can modulate oxidative stress responses and pro-inflammatory cytokine expression in T cells and dendritic cells, suggesting its potential role in immune regulation. Moreover, *NSUN2*-dependent methylation of the transcription factor interferon regulatory factor 4 (IRF4) can reshape the immune phenotype of macrophages from M1 to M2 macrophage adhering to titanium implant surfaces, thereby influencing the osteogenic and angiogenic capacity of the surrounding microenvironment.^65^ Beyond *NSUN2*, other m^5^C regulators have also been implicated in immune-related pathologies. For instance, Tet methylcytosine dioxygenase 2 (*Tet2*) has also been shown to promote M2 macrophage polarization in allergic rhinitis through m^5^C modifications on mRNA, indicating its involvement in tissue-specific inflammatory responses.^66^

Collectively, these findings highlight the emerging significance of m^5^C modifications in shaping macrophage functional diversity. Nevertheless, the roles of *NSUN2* and other m^5^C-related enzymes in intestinal macrophages remain poorly understood. Given their potential regulatory impact in the context of chronic inflammation and gut microbiota dysbiosis, further investigation is warranted to elucidate their contributions to intestinal immune homeostasis.

## Emerging insights into m^1^A modification in macrophages

m^1^A is a distinct post-transcriptional RNA modification predominantly found in tRNAs and rRNAs, but it also occurs in mRNA, especially within the 5′ untranslated region (5′ UTR) and near the start codon. m^1^A is thought to influence RNA secondary structure and enhance translational efficiency. The installation of m^1^A is catalyzed by specific methyltransferases, such as tRNA methyltransferase 6 (*TRMT6*)*/TRMT61A* for mRNA and *TRMT10C* for mitochondrial RNA, while its removal is mediated by demethylases, including *ALKBH1* and *ALKBH3*. *YTHDF* family proteins, especially *YTHDF3*, are considered potential readers of m^1^A in mammalian cells, although their binding specificity is still under investigation.^67^^,^^68^

Compared with m^6^A, the role of m^1^A in immune cells remains less defined. However, recent studies have begun to shed light on its involvement in macrophage polarization and functional regulation. For example, a study on abdominal aortic aneurysm demonstrated that m^1^A modification, recognized by *YTHDF3*, regulates macrophage polarization and may contribute to inflammatory vascular pathology.^69^ Additionally, a novel class of tRNA-derived fragments (tRFs), such as tRF-3022b, has been shown to interact with specific cytokines in colorectal cancer, regulating tumor-associated apoptosis and M2 macrophage polarization. The expression of these tRFs is influenced by m^1^A-related factors, including *ALKBH3*, DNA methyltransferase 2 (*DNMT2*), angiogenin (*ANG*), and argonaute 2 (*AGO2*), suggesting a broader regulatory network involving m^1^A in macrophage functional reprogramming.^70^

Further evidence comes from single-cell and spatial transcriptomics studies. One investigation constructed a risk model based on m^1^A-associated microRNAs, revealing a strong correlation with immune cell infiltration in hepatocellular carcinoma.^71^ Another spatial transcriptomic analysis identified *ALKBH1* as a spatially associated factor with tumor-associated macrophages in gastric cancer tissues.^72^

Among the identified demethylases, *ALKBH3* is currently the only one demonstrated to directly remove m^1^A marks, particularly from tRNA and mRNA substrates. Although no direct studies have examined the role of *ALKBH3* in intestinal inflammation, such as inflammatory bowel disease, our group is currently conducting a systematic investigation using myeloid-specific conditional knockout mouse models to evaluate the function of *ALKBH3* in gut immune regulation. These studies aim to broaden our understanding of m^1^A modification in maintaining immune homeostasis and provide a theoretical basis for RNA modification-based therapeutic strategies.

Taken together, these findings indicate that RNA modifications regulate macrophage plasticity not as isolated switches but as integral components of broader signaling and transcriptional networks. Writers, erasers, and readers of m^6^A, m^5^C, and m^1^A converge on canonical pathways such as nuclear factor-kappa B (NF-κB), Janus kinase/signal transduction and transcription activation (JAK–STAT), and metabolic checkpoints including glycolysis and FAO/oxidative phosphorylation (OXPHOS), thereby rewiring transcription factor programs (*e.g.*, STAT1/STAT6, IRFs, PPARγ) that shape macrophage identity (Table 1). Such interactions highlight that the epitranscriptome operates as a nodal layer linking environmental cues to transcriptional outcomes, enabling macrophages to flexibly transition between pro-inflammatory, reparative, and tolerogenic states. This integrative perspective sets the stage for understanding how tissue-specific contexts, particularly the inflammatory and microbial environment of the gut, further remodel these RNA-mediated regulatory circuits.

**Table 1:** Representative RNA modification regulators, mechanistic targets, and their roles in macrophage polarization and intestinal disease contexts.

Modifier	Category	Modification	Targets/pathways	Functional effect in macrophages	Disease (relevance)
*METTL3*	Writer	m^6^A	*STAT1, Pyk2, HDGF*	Promotes M1 polarization; enhances glycolysis and pro-inflammatory programs	Atherosclerosis, IBD, CRC
*FTO*	Eraser	m^6^A	*STAT1, PPARγ*	Modulates both M1 and M2 programs depending on context	Metabolic inflammation, cancer
*ALKBH5*	Eraser	m^6^A	*CPT1A, SOCS2, IL-11*	Stabilizes FAO genes, promotes M2-like/tissue-repair phenotypes; regulates macrophage-to-myofibroblast transition	CRC, lung cancer, cardiac fibrosis
*YTHDF2*	Reader	m^6^A	*TNF, JAK2/STAT1* transcripts	Degrades inflammatory mRNAs, restrains excessive activation	Sepsis, colitis
*IGF2BP2*	Reader	m^6^A	*PPARγ, TSC1, CPT1A*	Stabilizes metabolic regulators, drives OXPHO*S*-dependent M2 polarization	Tumor immunity, tissue repair
*NSUN2*	Writer	m^5^C	*IRF3, IRF4*	Represses type I IFN via *IRF3*; modulates M2 polarization via *IRF4*	Viral infection, tissue remodeling
*TET2*	Writer/demethylase	m^5^C	Multiple mRNAs (*e.g.*, cytokines)	Promotes M2 polarization in allergic inflammation	Allergic rhinitis, chronic inflammation

## Epitranscriptomic regulation of intestinal mononuclear phagocytes in the gut microenvironment

**Figure 3:**
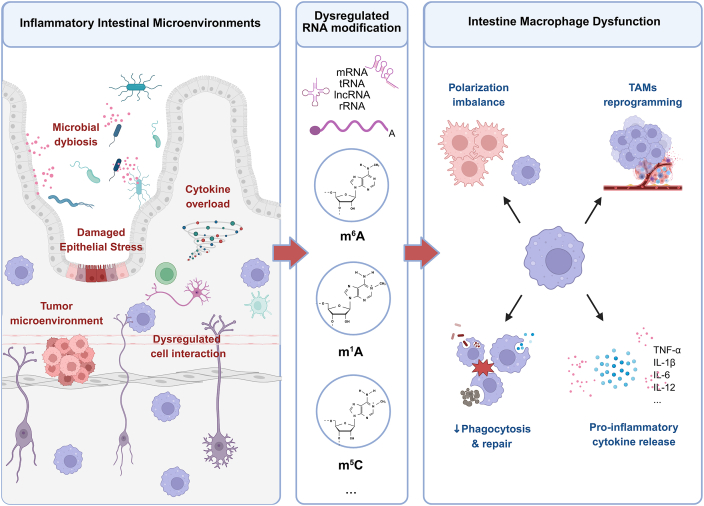
Inflammatory microenvironments reshape RNA-mediated regulation of intestinal macrophage. In inflammatory conditions of the gut, including infection, tissue damage, and tumor-associated inflammation, external stimuli, such as microbial dysbiosis, cytokine overload, and epithelial stress, modulate the expression and activity of RNA-modifying enzymes. Disrupted m^6^A/m^1^A/m^5^C modification dynamics in intestinal macrophages affect mRNA stability, translation, and degradation of immune-related transcripts. These epitranscriptomic changes contribute to aberrant macrophage polarization, excessive inflammatory signaling, and impaired resolution of inflammation. m^6^A, N^6^-methyladenosine; m^1^A, N^1^-methyladenosine; m^5^C, 5-methylcytosine; lncRNA, long non-coding RNA; rRNA, ribosomal RNA; TAM, tumor-associated macrophage.

Although RNA modifications are intracellular molecular events, their expression and functions are profoundly influenced by signals from the tissue microenvironment. Studies have revealed that the expression patterns of RNA-modifying enzymes vary among macrophages from different tissue origins, suggesting that external cues, such as microbial metabolites and local inflammatory mediators, may regulate the expression or activity of these enzymes, thereby indirectly shaping macrophage phenotypes and functions. In the highly dynamic and complex gut environment, the intestinal microbiota, microbial metabolites, physical barrier structures, and chronic inflammatory stimuli together constitute a multidimensional signaling network. This network may reshape the RNA modification landscape of intestinal macrophages, ultimately influencing their roles in maintaining homeostasis, supporting barrier integrity, and modulating immune responses. Although direct studies on the epitranscriptomic regulation of intestinal mononuclear phagocytes remain limited, substantial evidence has demonstrated the pivotal role of RNA modifications in regulating macrophage fate and function. In the following sections, we will discuss how inflammatory microenvironments, microbial metabolites, and intercellular interactions modulate RNA modifications in intestinal macrophages (Fig. 3).

## The impact of the inflammatory gut microenvironment on macrophage fate

m^6^A methylation is widely involved in the pathogenesis and progression of inflammatory bowel disease, and extensive alterations in m^6^A modifications have been observed in intestinal tissues from patients with inflammatory bowel disease.^73^ The m^6^A “reader” protein *YTHDF1* is also involved in regulating the NF-κB pathway in macrophages by promoting the translation of tumor necrosis factor receptor-associated factor 6 (TRAF6), thereby enhancing inflammatory responses.^74^ More directly, loss of the nuclear m^6^A reader *YTHDC1* in intestinal macrophages exacerbates dextran sodium sulfate-induced colitis. *YTHDC1* exerts anti-inflammatory effects by stabilizing Ras homolog family member H (RHOH) to suppress pro-inflammatory signaling, while concurrently enhancing epithelial barrier function by regulating nucleoside diphosphate kinase 1 (NME1).^75^ In addition, human umbilical cord-derived mesenchymal stem cell exosomes (hucMSC-Ex) have been shown to alleviate colitis in mice by enhancing M2 macrophage polarization via the *METTL3*–solute carrier family 37 member 2 (Slc37a2)–YTHDF1 axis, thereby suppressing pro-inflammatory macrophage activity.^76^

During the remission phase of inflammatory bowel disease, the distribution and compartmentalization of intestinal macrophages also undergo significant changes.^77^ These cells are crucial for epithelial regeneration following injury^78^^,^^79^ and can be reprogrammed into regulatory phenotypes by therapies such as anti-TNF-α antibodies, contributing to the resolution of inflammation.^80^ However, how RNA modifications regulate the function of intestinal mononuclear phagocytes during inflammatory bowel disease remission remains largely unclear and warrants further investigation.

## Microbial regulation of RNA modifications in intestinal mononuclear phagocytes

The early development of the intestinal immune system is highly dependent on the gut microbiota,^81^ and the replenishment and recruitment of intestinal macrophages are likewise influenced by microbial cues.^2^^,^^24^^,^^82^ Gut microorganisms regulate immune responses, metabolic activities, and epithelial barrier function by producing metabolites such as bile acids and short-chain fatty acids, thereby maintaining intestinal homeostasis and systemic immune balance.^83^ Together with the epithelium, these microbes protect the host from pathogens through coordinated metabolic and signaling processes that promote mucosal health. Notably, the microbiota also modulates the local hypoxic environment of the gut, which is essential for nutrient absorption, epithelial function, and mucosal immunity.^84^

Accumulating evidence indicates that the gut microbiota can influence host RNA epigenetic modifications.^85^^,^^86^ In germ-free mice, the intestinal m^6^A landscape is significantly altered.^87^^,^^88^ Specific microbes, such as *Saccharomyces boulardii*, *enterotoxigenic Escherichia coli K88 (E. coli K88)*, and *Salmonella Typhimurium* (HKST), can affect both m^6^A modification patterns and the transcription of m^6^A regulators in intestinal tissues.^89^, ^90^, ^91^ These effects may be mediated via microbial metabolites or toxins. For instance, aflatoxin B1 (AFB1) exposure results in substantial alterations in the m^6^A modification of genes related to the cell cycle, endoplasmic reticulum function, and mitophagy.^92^ Additionally, microbiota-derived metabolites, such as betaine, folate, and cyclic leucine, have been shown to influence immune cell function through epigenetic pathways.^93^, ^94^, ^95^ Conversely, RNA modifications can also impact gut microbial homeostasis.^85^^,^^96^ *METTL14* deficiency not only induces spontaneous colitis in mice but also alters gut microbiota composition at 24 weeks.^87^ *YTHDF1* knockout has been shown to improve gut fungal diversity and probiotic colonization three days after traumatic brain injury in mice.^97^

Current studies exploring the regulation of RNA modifications in intestinal mononuclear phagocytes by the gut microbiota remain limited. Previous research has shown that *Bacteroides fragilis* toxin suppresses *METTL3*-mediated m^6^A methylation in macrophages, thereby promoting intestinal inflammation and exacerbating inflammatory bowel disease.^50^ Additionally, lipopolysaccharide produced by Gram-negative bacteria has been found to alter m^6^A modification patterns in intestinal macrophages. Upon lipopolysaccharide stimulation, the expression of *METTL3* and *YTHDF2* is significantly reduced, and silencing either factor enhances lipopolysaccharide-induced inflammatory responses through up-regulation of pro-inflammatory mediators and activation of NF-κB signaling.^98^^,^^99^ Nevertheless, the precise mechanisms by which the gut microbiota modulates RNA modifications in mononuclear phagocytes require further investigation.

## Crosstalk with neighboring cells influences RNA modifications in macrophages

The maintenance of intestinal immune homeostasis relies heavily on intercellular communication. Intestinal mononuclear phagocytes, particularly macrophages, are not only regulated by neighboring epithelial, neuronal, and immune cells but also actively influence the development, differentiation, and function of these cells through secreted factors, cell–cell contact, and metabolic signaling.

Intestinal macrophages are closely associated with the intestinal epithelium and play a critical role in preserving barrier integrity. They promote epithelial renewal and remodeling by secreting factors, such as Wnts, PGE_2_, and hepatocyte growth factor (HGF), and facilitate epithelial repair through IL-10-mediated induction of WNT1-inducible signaling protein 1 (WISP1).^100^ In addition, macrophages help maintain homeostasis by clearing apoptotic epithelial cells.^78^^,^^101^, ^102^, ^103^

Conversely, epithelial cells also influence the differentiation of intestinal mononuclear phagocytes.^104^ They express ligands such as Notch pathway components and CX3CL1 to guide macrophage development^105^^,^^106^ and release soluble factors that modulate macrophage phenotype.^107^ CSF1R-dependent macrophages are essential for epithelial stem cell maintenance. Blocking CSF1R leads to macrophage depletion, resulting in impaired Paneth cell differentiation and a reduced population of leucine-rich repeat-containing G protein-coupled receptor 5-positive (Lgr5^+^) intestinal stem cells.^2^

Macrophages also form extensive perivascular networks in the intestinal mucosa,^108^ supporting endothelial integrity by producing vascular endothelial growth factors (*e.g.*, VEGF-C).^109^ Additionally, they interact with the enteric nervous system (ENS) through the secretion of TGF-β family members such as bone morphogenetic protein 2 (BMP2) and cytokines like resistin-like molecule-α (RELMα), providing metabolic and trophic support.^110^^,^^111^

The bidirectional interaction between macrophages and the ENS is particularly critical. During ENS development, muscularis-resident macrophages contribute to the establishment and structural maintenance of enteric neurons.^112^ Upon ENS maturation, TGF-β signaling drives the differentiation of nerve-associated macrophages, which are distributed throughout the myenteric plexus and remain in close contact with enteric neurons. With aging, macrophages tend to adopt a pro-inflammatory phenotype, contributing to ENS degeneration.^113^ Although ENS is not required for the initial development of intestinal macrophages,^114^ enteric neurons can modulate macrophage activity via norepinephrine signaling, enhancing their tissue-protective function.^111^ Moreover, the ENS is a primary source of colony-stimulating factor 1 (CSF1), a key growth factor for mononuclear phagocyte differentiation and maintenance.^110^ Interestingly, activation of transient receptor potential cation channel subfamily V member 1-positive (Trpv1^+^) neurons originating from dorsal root ganglia significantly reduces macrophage numbers in the cecum and colon, suggesting that neurotransmitters contribute to the spatial regulation of macrophage homeostasis.^115^

RNA modifications, as important regulators of macrophage function, are also involved in modulating intercellular communication. For example, *YTHDC1* has been shown to enhance epithelial barrier integrity by regulating the expression of NME1.^75^ However, published studies in this area remain limited. In our unpublished data, we observed that macrophages deficient in Pcif1 promote the accumulation of extracellular matrix, thereby facilitating the maturation of choline acetyltransferase-positive (ChAT^+^) and neuronal nitric oxide synthase-positive (nNOS^+^) enteric neurons, ultimately attenuating pro-inflammatory responses and protecting neurons from inflammation-induced damage.

## Pathogenic associations and therapeutic potential of RNA modifications

RNA modification factors have demonstrated substantial pathogenic regulatory potential in various intestinal diseases. In inflammatory bowel disease, aberrant expression of *METTL3*, *YTHDC1*, and other modifiers in macrophages affects their polarization states, barrier-repair capacity, and inflammatory responses, positioning them as critical regulators of immune homeostasis. In infectious colitis models, pathogen-associated signals such as lipopolysaccharide can down-regulate *METTL3* and *YTHDF2*, thereby exacerbating inflammation, suggesting a role for RNA modifications in tuning antimicrobial immune responses. Within the colorectal cancer microenvironment, tumor-associated macrophages may rely on modifiers such as *IGF2BP1* to maintain an immunosuppressive phenotype. Targeted inhibition of these key enzymes, particularly *METTL3*, *ALKBH5*, and the *IGF2BP* family, has shown therapeutic potential in multiple models of inflammation and cancer, offering promising new avenues for the treatment of intestinal diseases.^116^, ^117^, ^118^

## Conclusions and future perspective

In recent years, RNA modifications have emerged as pivotal regulators of immune cell development and function, including in macrophages. As central mediators of mucosal immune surveillance and homeostasis, intestinal mononuclear phagocytes, particularly macrophages, require precise environmental cues for functional adaptation. RNA modifications may represent a critical layer of post-transcriptional regulation that enables such dynamic responsiveness.

Accumulating evidence suggests that modifications such as m^6^A orchestrate macrophage polarization, cytokine production, and tissue repair capacity, contributing to the pathogenesis of intestinal diseases, such as inflammatory bowel disease, infectious colitis, and colorectal cancer. The unique gut microenvironment, shaped by microbial metabolites, epithelial-derived signals, and neuroimmune interactions, likely remodels RNA modification landscapes in intestinal macrophages, thereby influencing their immunological behavior.

Despite these insights, our current understanding of RNA modifications in intestinal macrophages remains limited. High-resolution, *in situ* maps of modification dynamics are lacking, and the functional heterogeneity of modification enzymes across macrophage subsets is yet to be fully characterized. Most therapeutic efforts targeting RNA modifications have thus far focused on cancer models, leaving their translational relevance in mucosal inflammation relatively unexplored.

In addition, other RNA modifications, such as N4-acetylcytidine (ac^4^C) and pseudouridine (Ψ), have scarcely been investigated in intestinal macrophages. While N-acetyltransferase 10 (*NAT10*)-mediated ac^4^C has been linked to macrophage inflammation,^119^ and pseudouridylation is known to modulate innate RNA sensing and colorectal cancer progression,^120^ direct evidence in intestinal mononuclear phagocytes is still lacking. Of note, our group has recently shown that *NAT10*-dependent ac^4^C modification sustains T cell pathogenicity in inflammatory bowel disease, underscoring the relevance of this pathway to mucosal immunity.^121^ Ongoing work in our laboratory is also exploring whether *NAT10*-mediated ac^4^C and related modifications exert analogous regulatory functions in intestinal macrophages. Future studies that address these less characterized modifications will be essential to provide a more complete picture of epitranscriptomic regulation in gut immunity.

Future studies should focus on delineating the spatial and temporal regulation of RNA modifications in intestinal macrophage subsets. Integrative approaches combining single-cell transcriptomics, methylated RNA immunoprecipitation sequencing, spatial transcriptomics, and *in situ* modification imaging will be essential to construct high-resolution modification atlases. Functional interrogation using organoid systems, conditional knockout mice, and humanized models will help elucidate causal links between specific modifications and macrophage phenotypes. Furthermore, the regulatory influence of gut-derived factors, such as microbiota, epithelial cues, and neurotransmitters, on RNA-modifying enzymes warrants deeper investigation. Ultimately, the development of selective modulators targeting RNA writers, erasers, or readers holds promise for reprogramming intestinal macrophage function in inflammatory bowel disease, infectious inflammation, and colorectal malignancies.

Collectively, these efforts will not only deepen our mechanistic understanding of RNA epitranscriptomic control in gut immunity but also open new avenues for precision immunotherapy targeting intestinal mononuclear phagocytes.

## CRediT authorship contribution statement

**Manqiqige Su:** Writing – original draft, Methodology, Investigation, Formal analysis. **Jiyuan Fan:** Writing – review & editing. **Hua-Bing Li:** Writing – review & editing, Conceptualization.

## Data availability

Data sharing is not applicable to this article as no new data were created or analyzed in this study.

## Funding

This work was supported by the National Natural Science Foundation of China (82341017, 82030042, 82325024, 82350112, 82461160323, 82441048 to Hua-Bing Li), the National Key R&D Program of China (2025YFC3410100 and 2021YFA1100800 to Hua-Bing Li), and Shanghai Municipal Health Commission of China (2022XD047 and 2022JC0 to Hua-Bing Li).

## Conflict of interests

No potential conflict of interests was reported by the authors.
